# In vitro models to detect in vivo bile acid changes induced by antibiotics

**DOI:** 10.1007/s00204-022-03373-4

**Published:** 2022-09-08

**Authors:** Nina Zhang, Jingxuan Wang, Wouter Bakker, Weijia Zheng, Marta Baccaro, Aishwarya Murali, Bennard van Ravenzwaay, Ivonne M. C. M. Rietjens

**Affiliations:** 1 Division of Toxicology, Wageningen University and Research, Stippeneng 4, 6708 WE Wageningen, The Netherlands; 2 BASF SE, 67056 Ludwigshafen, Germany

**Keywords:** Antibiotics, Tobramycin, fecal incubations, Bile acid homeostasis, 16S rRNA analysis, Bile acid reuptake

## Abstract

Bile acid homeostasis plays an important role in many biological activities through the bile–liver–gut axis. In this study, two in vitro models were applied to further elucidate the mode of action underlying reported in vivo bile acid changes induced by antibiotics (colistin sulfate, tobramycin, meropenem trihydrate, and doripenem hydrate). 16S rRNA analysis of rat fecal samples anaerobically incubated with these antibiotics showed that especially tobramycin induced changes in the gut microbiota. Furthermore, tobramycin was shown to inhibit the microbial deconjugation of taurocholic acid (TCA) and the transport of TCA over an in vitro Caco-2 cell layer used as a model to mimic intestinal bile acid reuptake. The effects induced by the antibiotics in the in vitro model systems provide novel and complementary insight explaining the effects of the antibiotics on microbiota and fecal bile acid levels upon 28-day in vivo treatment of rats. In particular, our results provide insight in the mode(s) of action underlying the increased levels of TCA in the feces upon tobramycin exposure. Altogether, the results of the present study provide a proof-of-principle on how in vitro models can be used to elucidate in vivo effects on bile acid homeostasis, and to obtain insight in the mode(s) of action underlying the effect of an antibiotic, in this case tobramycin, on bile acid homeostasis via effects on intestinal bile acid metabolism and reuptake.

**Supplementary Information:**

The online version contains supplementary material available at 10.1007/s00204-022-03373-4.

## Introduction

Bile acids play a key role in absorbing intestinal nutrients, emulsifying lipids, and secreting toxic metabolites and xenobiotics. Additionally, bile acids are important signaling molecules that are able to modulate lipid, glucose, and energy metabolism by reacting with diverse bile acid receptors (Houten et al. 2006; Thomas et al. 2008).

**Fig. 1:**
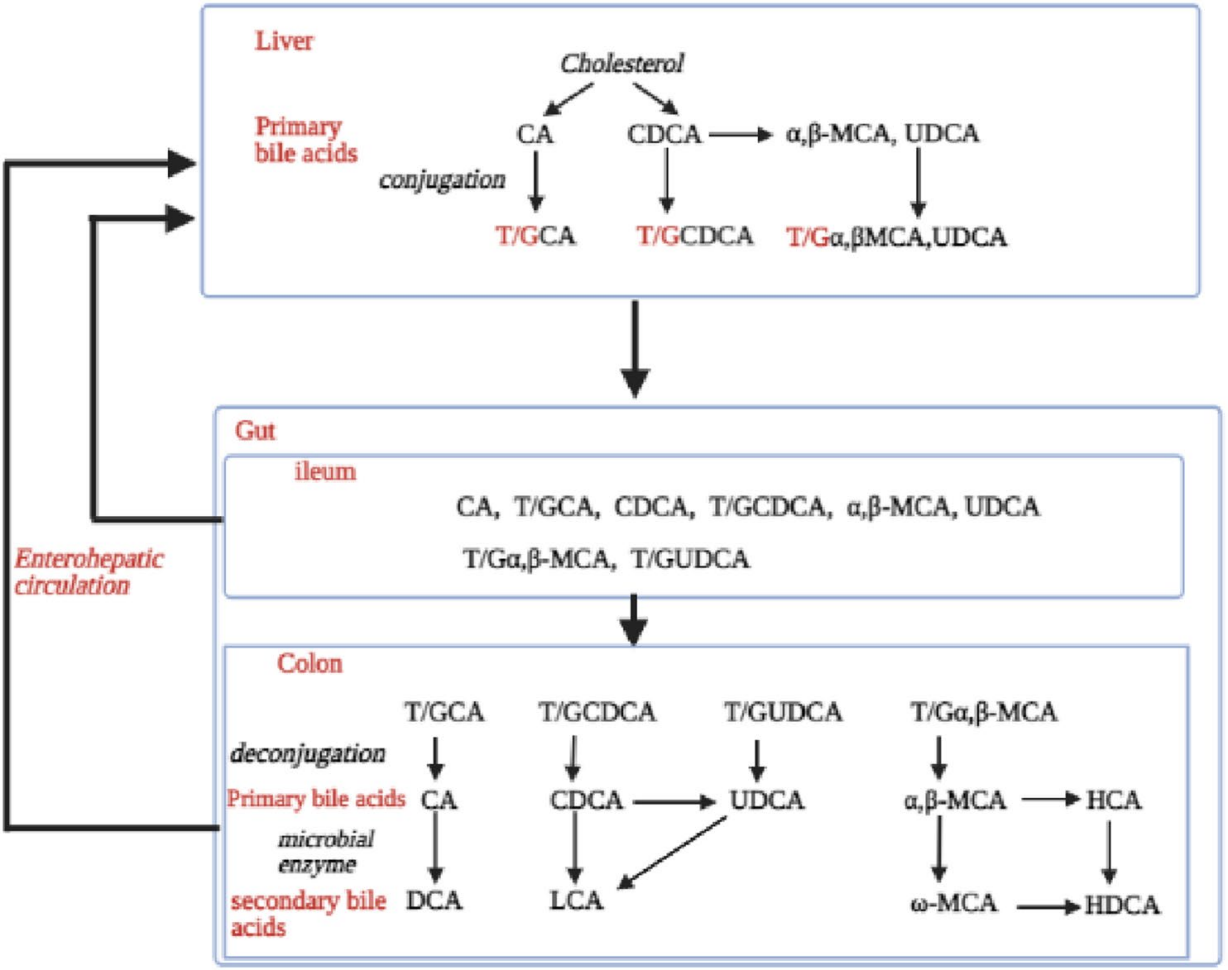
Bile acid synthesis and conversion. The primary bile acids α-MCA and β-MCA and their secondary bile acids are rodent specific

Cholic acid (CA) and chenodeoxycholic acid (CDCA) are the two primary bile acids synthesized from cholesterol in the liver and further conjugated with taurine or glycine to generate taurocholic acid (TCA), glycocholic acid (GCA), taurochenodeoxycholic acid (TCDCA), and glycochenodeoxycholic acid (GCDCA) increasing their solubility (Di Ciaula et al. 2018; Stamp and Jenkins 2008). Conjugated bile acids are secreted into the duodenum after a meal through the bile duct (Chiang and Ferrell 2018). Upon release into the intestinal tract typically, 95% of bile acids are actively reabsorbed in the ileum of the small intestine, resulting in enterohepatic circulation (Chiang 2009; Ridlon et al. 2016). A minor amount of bile acids escapes this reabsorption and enters the colon where the indigenous gut microbiota processes their deconjugation, dehydrogenation, and dihydroxylation converting the primary bile acids into secondary bile acids, such as deoxycholic acid (DCA), lithocholic acid (LCA), and the tertiary Bas, including, for example, ω-muricholic acid (ω-MCA) (Winston and Theriot 2020). Ursodeoxycholic acid (UDCA) is the primary bile acid in rodents being the 7β-hydroxy isomer of CDCA which is also converted to UDCA and subsequently to LCA by intestinal bacteria (Ridlon and Bajaj 2015). An overview of bile acid synthesis and conversion is presented in Fig. 1. Disturbance of bile acid homeostasis has been related to several metabolic diseases, such as obesity, type 2 diabetes mellitus, and inflammatory bowel disease (IBD) (Duboc et al. 2013; Jones et al. 2014). In previous studies, it was shown that in vivo oral exposure to antibiotics may affect bile acid homeostasis resulting in changes in the bile acid metabolic profiles in intestinal tissues, feces, and blood samples (Behr et al. 2018b, 2019), . These effects were (in part) related to the effects of antibiotics on the host intestinal microbiota, known to contribute to bile acid metabolism.

The intestinal microbiota consists of approximately 10^14^ microbes including 500 to 1000 distinct bacterial species (Eckburg et al. 2005). It is estimated that gut microbiota includes more than 1000 phylotypes dividing into six phyla: *Firmicutes*, *Bacteroidetes*, *Proteobacteria*, *Actinobacteria*, *Fusobacteria*, and *Verrucomicrobia* (Consortium 2012; Wang et al. 2005). The metabolic activity of the intestinal microbiome is critical not only in modulating host immune defense but also in maintaining host metabolism and health, as illustrated for example by studies using germ-free animals (Claus et al. 2008; Wikoff et al. 2009).

The gut microbiota participates in many metabolic processes such as vitamin synthesis, regulating dietary lipid metabolism, indigestible carbohydrates metabolism and secondary bile acids syntheis. A lot of bile acid studies show that the bile acid pool size, composition, and compartment concentrations are directly linked to the constitution of the gut microbiota and the microbial metabolism of bile acids in the intestine (Sayin et al. 2013).

Antibiotics are able to alter the diversity of the gut microbiota, subsequently also changing the metabolome, especially altering the composition and concentration of bile acids (Antunes et al. 2011; Behr et al. 2018a; Yap et al. 2008). Vrieze and colleagues orally administered vancomycin to male subjects for 7 days and provided evidence that the antibiotics influence gut microbiota composition and the makeup and concentrations of bile acids (Vrieze et al. 2014). Kuno and colleagues observed changes in secondary bile acids [lithocholic acid (LCA) and deoxycholic acid (DCA)] in mice upon oral administration of the non-absorbable antibiotics vancomycin and polymyxin B (Kuno et al. 2018). Behr and colleagues observed that antibiotic treatment in rats caused differences in bile acid metabolome profiles in plasma and feces, especially significantly increasing taurine conjugated primary bile acids in both matrices (Behr et al. 2019). In recent studies, colistin sulfate and tobramycin which are also non-absorbable antibiotics appeared to also change the composition of the gut microbiota and influence bile acid metabolism, albeit to a different extent (Murali et al. in preparation).

In addition to effects on the microbiota, it might also be hypothesized that the effects of the antibiotics on the bile acid metabolome patterns may be related to an alteration of the bile acid reuptake into the liver in the ileum of the small intestine. Given that 95% of bile acids are actively reabsorbed, even a limited inhibition of the transporters involved can be expected to already substantially increase fecal levels of bile acids. The aim of the present study was to investigate if, and to what extent, the potential effects of antibiotics on in vivo bile acid homeostasis could be detected in in vitro model systems, and whether such in vitro studies could provide additional insights into the mode(s) of action underlying effects of the antibiotics on bile acid homeostasis.

To this end, we analyzed the bile acid changes in rat fecal incubations, with and without adding conjugated primary bile acids, upon treatment with antibiotics. 16S rRNA sequencing was performed to estimate the effects of the antibiotics on the intestinal microbiota abundances and LC–MS/MS was used to quantify bile acid patterns. Additionally, Caco-2 cell layers in a Transwell model were used to elucidate the potential impact of the studied antibiotics on the intestinal reuptake of bile acids.

## Materials and methods

### Chemicals and reagents

Chenodeoxycholic acid (CDCA), ursodeoxycholic acid (UDCA), lithocholic acid (LCA), hyodeoxycholic acid (HDCA), hyocholic acid (HCA), deoxycholic acid (DCA), cholic acid (CA), glycoursodeoxycholic acid (GUDCA), glycochenodeoxycholic acid (GCDCA), glycodeoxycholic acid (GDCA), glycocholic acid (GCA), taurolithocholic acid (TLCA), tauroursodeoxycholic acid (TUDCA), taurohyodeoxycholic acid (THDCA), taurochenodeoxycholic acid (TCDCA), taurodeoxycholic acid (TDCA), and taurocholic acid (TCA) were purchased from Sigma-Aldrich (Schnelldorf, Germany). Alpha-muricholic acid (α-MCA), beta-muricholic acid (β-MCA), and glycolithocholic acid (GLCA) were from Cambrige isotope laboratories (Massachusetts, USA). Tobramycin, colistin sulfate, meropenem trihydrate, and doripenem hydrate were purchased from Sigma-Aldrich (Schnelldorf, Germany). AcroPrep™ 96-well filter plates were purchased from Pall Corporation (Amsterdam, The Netherlands). Acetonitrile (ACN) and methanol were obtained from Biosolve BV (Valkenswaard, The Netherlands). Dimethyl sulfoxide (DMSO) was obtained from Sigma-Aldrich (Darmstadt, Germany). Phosphate-buffered saline (PBS) was purchased from Gibco (Paisley, UK). Fetal Bovine serum (FBS) was obtained from GE Healthcare Life Sciences Hyclone Laboratories (Logan, Utah, USA). 0.05% Trypsin–EDTA, minimum essential medium (MEM), penicillin–streptomycin-glutamine solution (PSG), sodium pyruvate, Hank’s balanced salt solution (HBSS), and HEPES buffer solution were purchased from Gibco (Paisley, UK). Roche cell proliferation reagent WST-1 was purchased from Sigma-Aldrich (Schnelldorf, Germany). Corning Costar 12-well Transwell plates were purchased from Corning Life Sciences (Schnelldorf, Germany). 96-Well cell culture plates were obtained from Greiner Bio-One B.V. (Alphen aan den Rijn, The Netherlands).

### Conversion of the in vivo dose levels to in vitro concentrations

To establish the in vitro concentrations of the antibiotics to be used in the in vitro studies, in vivo dose levels of the selected antibiotics (colistin sulfate, tobramycin, meropenem trihydrate, and doripenem hydrate) used in rat metabolomics studies at BASF (Murali et al. in preparation) were converted to corresponding in vitro test concentrations using Eq. 1

1 $$ \begin{gathered} {\text{In}}\;{\text{vitro}}\;{\text{test}}\;{\text{concentration}}\;{\text{(in}}\;{\text{mM)}} = ({\text{In}}\;{\text{vivo}}\;{\text{daily}}\;{\text{exposure}}\;{\text{of}}\;{\text{compound}}\;{\text{(in}}\;{\text{mg}}/{\text{kg}}\;{\text{bw)}} \hfill \\ \quad \times {\text{bw}}\;{\text{(in}}\;{\text{kg)}})/{\text{Volume}}\;{\text{of}}\;{\text{gastrointestinal}}\;{\text{tract}}\;{\text{(in}}\;{\text{mL)}}/{\text{Molecular}}\;{\text{weight}}\;{\text{of}}\;{\text{compound}}\;{\text{(mg}}/{\text{mmol)}} \hfill \\ \quad \times {1}000\;({\text{mL}}/{\text{L}}). \hfill \\ \end{gathered} $$

The in vitro test concentration thus derived from the in vivo dose levels are presented in Table S1 in the supplementary information. The volume of the gastrointestinal tract of rat was assumed to amount to 12 ml (McConnell et al. 2010), adding up the water content of a fed gastrointestinal tract of 10 g, and a solid content of 2 g, and assuming the density to be 1 g/ml. The concentrations used in the in vitro assays equaled the concentrations derived from the high-dose levels, unless stated otherwise.

### Anaerobic incubation of rat feces

#### Fecal slurry preparation

The fecal samples from tobramycin rats were provided by BASF and taken from an in vivo study in which Wistar rats were orally administrated a series of antibiotics, including tobramycin, for 28 days. The animal study was performed in an AAALAC-approved (Association for Assessment and Accreditation of Laboratory Animal Care International) laboratory in compliance with the German Animal Welfare Act and the effective European Council Directive. The study was approved by the BASF Animal Welfare Body, with the permission of the local authority, the Landesuntersuchungsamt Koblenz, Germany (approval number 23 177-07/G 18-3-098), and was carried out based on the OECD 407 Principles of Good laboratory Practice and the GLP provisions of the German Chemicals Act. Tobramycin was prepared in deionized water for oral administration at doses of 100 mg/kg body weight/day and 1000 mg/kg body weight/day, respectively. Fecal samples were taken from, respectively, control rats (10 males and 10 females) and exposed rats (5 males and 5 females) receiving 1000 mg/kg body weight/day tobramycin at day 23 of the experiment. Blood and fecal samples of the animals were collected for a detailed metabolomics study, the results of which will be described in a separate paper (Murali et al. in preparation). Feces were obtained by physical massage of the rectum of rats, weighed and transferred immediately into anaerobic 10% (v/v) glycerol in PBS solution, and pooled and diluted to a final fecal concentration of 20% (w/v) under an anaerobic atmosphere (85% N_2_, 10% CO_2_, and 5% H_2_) (BACTRON300 anaerobic chamber (Cornelius, USA)). Subsequently, samples were filtered using sterile gauze under anaerobic conditions, and aliquoted samples of resulting fecal slurry were stored at – 80 °C until use.

#### Treatment of fecal samples with antibiotics

To detect the effects of antibiotics on the composition of the fecal microbiota, 280 µl control rat fecal slurry mixed with 70 µl MilliQ water or 70 µl colistin sulfate, tobramycin, meropenem trihydrate, and doripenem hydrate (final concentration: 2 mM colistin sulfate, 45 mM tobramycin, 15 mM meropenem trihydrate, or 50 mM doripenem hydrate) were incubated under anaerobic conditions for 24 h at 37 °C. After 24 h, these samples were stored at – 80 °C overnight and shipped with dry ice for 16S rRNA analysis. Based on the results obtained (see “Results” section), tobramycin and colistin sulfate were selected for further studies.

For further studies with the selected antibiotics on effects on bile acid metabolism, Eppendorf tubes containing 80 µl control fecal slurry (final concentration: 160 mg feces/ml) and 20 µl tobramycin or 20 µl colistin sulfate solution to give final concentrations of 45 mM and 2 mM, respectively, were incubated under anaerobic condition; 80 µl fecal slurry and 20 µl MilliQ water served as control group. Incubations were performed in the BACTRON 300 anaerobic chamber (Sheldon, Cornelius, USA) with an atmosphere of 85% N_2,_ 10% CO_2_, and 5% H_2_, at 37 °C. At 0 h, 4 h, 8 h, and 24 h of incubation, the reaction was stopped by adding a similar volume (100 µl) of acetonitrile. Samples were subsequently sonicated for 5 min and centrifuged at 21,500*g* for 15 min at 4 °C. Then, the supernatants were transferred to a 96-well filter plate (Pall corporation, Amsterdam, The Netherlands) and filtered, and samples thus obtained were stored at − 80 °C overnight, followed by freeze drying for 8 h. The residuals thus obtained were dissolved in 100 µl (50 µl methanol: 50 µl water), and centrifuged at 21,500*g* for 15 min at 4 °C, after which the supernatants were transferred to LC–MS/MS vials for bile acids measurement by LC–MS/MS.

Because parallel in vivo results showed that fecal conjugated bile acids increased upon tobramycin treatment of rats (Murali et al. in preparation), in addition to incubations studying the effect of antibiotics on the bile acids present in the fecal samples as such, the effects of the antibiotics on externally added conjugated bile acid conversion by the intestinal microbiota were studied. To this end, anaerobic fecal incubations (total volume 100 µl) contained: 1 µl control fecal slurry (final concentration: 2 mg/ml fecal samples), 10 µl of a 10 times concentrated solution containing conjugated bile acids (TCA, TCDCA, GCA, or GCDCA) resulting in a final concentration of 500 µM for each of the bile acids tested, 79 µl PBS and 10 µl water (control) or 10 µl tobramycin or colistin sulfate from 10 times concentrated stock solutions in MilliQ water resulting in final concentrations of 45 mM and 2 mM, respectively. Fermentation Eppendorf tubes were incubated in the BACTRON 300 anaerobic chamber (Sheldon, Cornelius, USA) with an atmosphere of 85% N_2,_ 10% CO_2_, and 5% H_2_, at 37 °C. Incubations were ended after 0 h, 2 h, 4 h, 6 h, and 8 h, by adding a similar volume (100 µl) of acetonitrile. Samples were subsequently centrifuged at 21,500*g* for 15 min at 4 °C, and the supernatants thus obtained were transferred to LC–MS/MS vials to store at − 80 °C until bile acids measurement by LC–MS/MS.

Furthermore, also incubations of fecal samples from tobramycin-treated rats with TCA (final concentration: 500 µM) were performed and processed in a similar way.

### 16S rRNA gene sequencing analysis

Fecal samples were sent to an accredited commercial laboratory (IMGM Laboratories GmbH, Martinsried, Germany) for DNA extraction, PCR, library preparation, and sequencing. Besides, quantification of the bacterial load was implemented by real-time qPCR. 16S V3–V4 primers (F-NXT-Bakt-341F: 5′-CCTACGGGNGGCWGCAG-3′ and R-NXT-Bakt-805R: 5′-GACTACHVGGGTATCTAATCC-3′) were used to amplify the PCR products. During an index PCR, barcodes for multiplexed sequencing were introduced using overhang tags. A sequencing library was prepared from barcoded PCR products and sequenced on the Illumuna MiSeq next-generation sequencing system (Illumuna Inc.). Signals were processed to *.fastq-files and the resulting 2 × 250 bp reads were demultiplexed. Microbiota identification were performed by clustering the operational taxonomic units (OUT).

### Caco-2 cell transport experiment

#### Cell cultures

Caco-2 cells were purchased from the American Type Culture Collection (Rockville, MD, USA). Passage 10–20 of Caco-2 cells were applied in this study. The cells were maintained in a culture medium consisting of MEM with 20% FBS, 1% sodium pyruvate, and 1% penicillin–streptomycin–glutamine. The cells were kept in an incubator at 37 °C, 5% CO_2_, and 100% humidity. The cells were passaged twice each week upon detachment with trypsin–EDTA (0.05%).

#### WST-1 assay

Caco-2 cells were cultured in 75 cm^2^ flasks in an incubator at 37 °C, 5% CO_2_ and 100% humidity to allow the cells to grow. Cells were collected from the flask at 50–60% confluence and seeded in the inner wells of a 96-well plate by adding 100 µl per well of a cell suspension containing of 4 × 10^5^/ml cells. To limit evaporation, 100 µl PBS was added to the outer wells. The plate was incubated for 18 days in the incubator at 37 °C, 5% CO_2_ and 100% humidity to allow the cells to attach and differentiate, changing the medium-to-fresh medium every other day. After 18 days in culture, cells were exposed for 48 h to solvent control (0.5% DMSO in MEM medium), tobramycin (final concentrations: 10 µM, 100 µM, 1 mM, 5 mM, 10 mM, 20 mM, 45 mM, or 100 mM directly prepared in MEM medium with 0.5% DMSO), and positive control (10 mM potassium dichromate added to MEM from a 200 times concentrated stock solution in DMSO resulting in 0.5% DMSO and 50 µM final concentration). After 48 h, 5 µl WST-1 solution were added to each well and after 2 h further incubation, and the absorbance was measured with a Softmax Pro 7.1 (California, US) at 440 nm and 620 nm. Data were acquired by deducting the 620 nm signal from the 440 nm signal and expressing the value for the treated samples as percentage of the control set at 100% viability.

#### The effect of tobramycin on TCA transport over a Caco-2 cell layer

To quantify the effect of tobramycin on transport of bile acids over an intestinal cell model, 0.5 ml of Caco-2 cell suspension in MEM medium containing 4 × 10^5^ cells/ml were seeded in the apical chambers of a Corning 12-well Transwell plate, while 1.5 ml MEM medium was added to the basolateral chambers. The Transwell plate was incubated for 18 days at 37 °C, 5% CO_2_ and 100% humidity to allow the cells to differentiate into a confluent cell layer. Medium was refreshed every other day. After this 18-day differentiation period, cells were exposed to 45 mM tobramycin for 48 h. The TEER values of the cell layer were measured using a Millicell® ERS-2 Volt-Ohm Meter (Millipore, Amsterdam, The Netherlands) and this was also done after exposure to tobramycin to confirm the integrity of the cell layer. Upon 48 h exposure to tobramycin, MEM medium was replaced by transport medium (HBSS supplemented with 10 mM HEPES solution) and the cells were incubated for 30 min, after which medium in the apical compartment was replaced with 0.5 ml transport medium containing 5 µM TCA (added from a 1 mM concentrated stock solution in DMSO) and medium in the basolateral compartment was replaced with 1.5 ml transport medium. Next, the cells were incubated, taking 75 µl samples from the basolateral compartment at 1 h, 2 h, and 3 h, refilling the volume with 75 µl transport medium.

### Bile acid profiling by LC–MS/MS analysis

Bile acid analysis was performed on a triple quadrupole LC–MS/MS system, model LCMS-8045 (Shimadzu Corporation, Japan), using a method able to measure 20 bile acids: UDCA, HDCA, CDCA, and DCA with values of Q1/Q3: 391.3/391.3; β-MCA, α-MCA, HCA, and CA with values of Q1/Q3: 407.3/407.3; GLCA with values of Q1/Q3: 432.3/74; GUDCA, GDCA, and GCDCA with values of Q1/Q3: 448.3/74; GCA with values of Q1/Q3: 464.3/74; TUDCA, THDCA, TCDCA, and TDCA with values of Q1/Q3: 498.4/498.4; TCA with values of Q1/Q3: 514.4/514.4; TLCA with values of Q1/Q3: 482.3/482.3; and LCA with values of Q1/Q3: 375.3/375.3. Bile acids in fecal samples and standards were separated on an Kinetex C18 column (1.7 µm × 100 A × 50 mm × 2.1 mm, Phenomenex 00B-4475-AN) using an ultra-high performance liquid chromatography (UHPLC) system (Shimadzu) with gradient elution using MilliQ water (0.01% formic acid) and methanol/acetonitrile (50% v/50% v) as mobile phase A and B, respectively. To enhance chromatographic performance, a C18 2.1 mm security guard (Phenomenex AJ0-8782) precolumn was used. Samples were injected (1 µl) onto the column equilibrated in 30% B at a flow rate of 0.4 ml/min. The following gradient was used: 0–10 min 30–70% B, 11–19 min 70–98% B and then 20–25 min 98–30% B with 10 min equilibration at 30% B before the next injection. The column temperature was set at 40℃ and the sample tray temperature was set at 4 °C. The mass spectrometer (MS) used electrospray ionization (ESI) in negative-ion mode. The ESI parameters were as below: nebulizing gas flow, 3 l/min; drying gas flow and heating gas flow, 10 l/min; interface temperature, 300 °C; disolvation temperature, 526 °C; heat block temperature, 400℃. Selective ion monitoring (SIM) and multiple reaction monitoring (MRM) were used for the detection of the bile acids.

### Data analysis

Metabolic profile data acquisition and processing were implemented using the Labsolutions software in the LC–MS/MS system. Graphics were drawn using Graphpad Prism 5 (San Diego, USA). Statistical analysis was performed by one-way analysis of variance with Dunnett’s post hoc test, or Student’s *t* test. *P* < 0.05 was considered statistically significant. Results are shown as mean ± standard deviation (SD). The bile acid figure was drawn using Biorender (San Francisco, USA). 16S rRNA analysis data were analyzed with R version 3.6.1 and QIIME 2 view.

## Results

### 16S rRNA microbial profile and correlation with metabolite formation

**Fig. 2:**
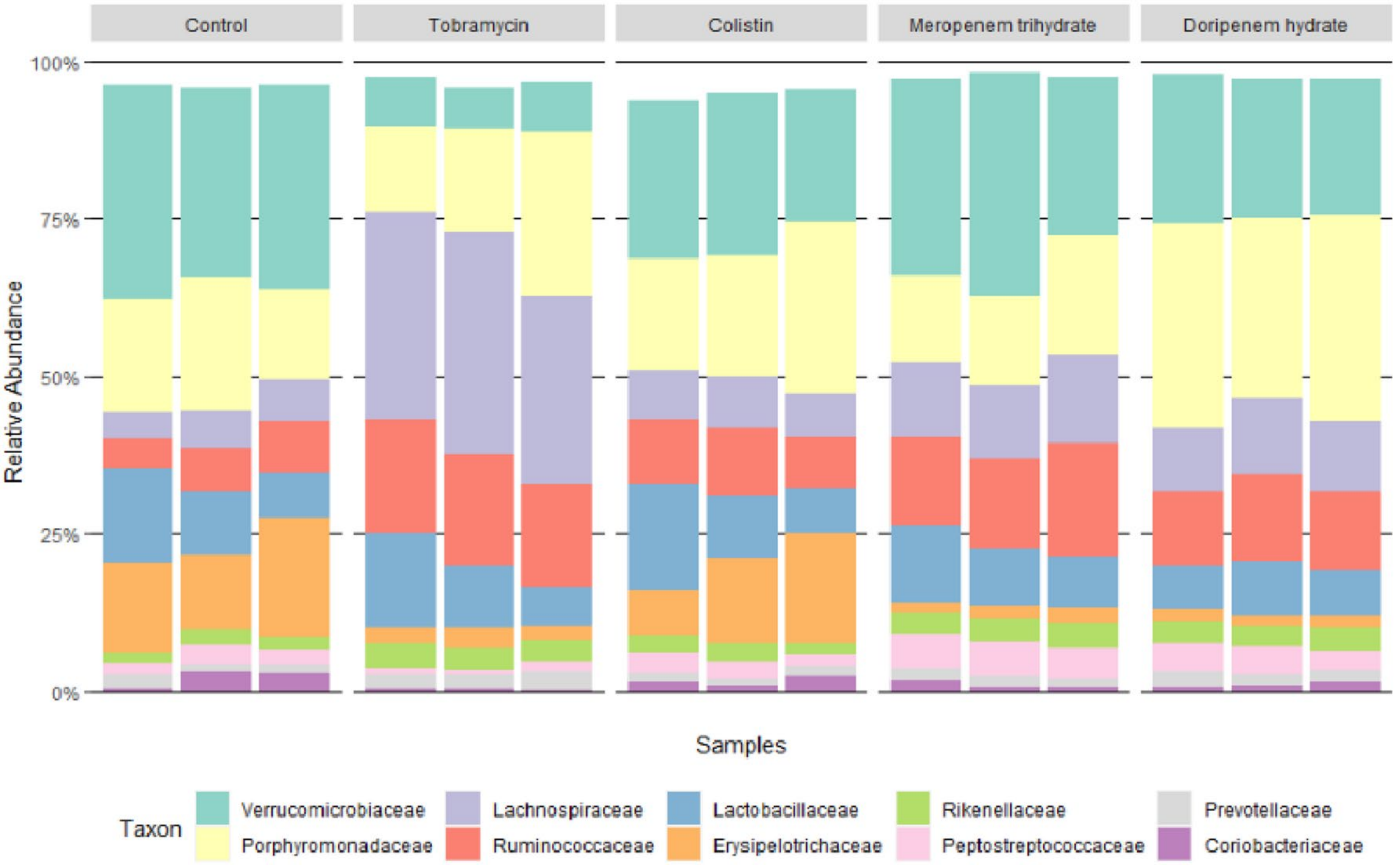
Relative microbial abundance at the dominant family level of the 24 h rat fecal samples from control rats either without (control) or with in vitro antibiotic treatment

Figure 2 presents the results of the 16S rRNA analysis of the gut microbiota composition of the fecal samples from control rats incubated in vitro either without (control) or with antibiotics (tobramycin, colistin sulfate, meropenem trihydrate, and doripenem hydrate), showing the relative microbial profile for dominant families. The untreated in vitro fecal microbial community appeared to consist mainly of *Verrucomicrobiaceae*, followed by *Porphyromonadaceae* and *Erysipelotrichaceae*, *Lactobacillaceae*, *Ruminococcaceae*, and *Lachnospiraceae*. Comparing the microbial communities in the control group to those observed for the fecal samples treated with the antibiotics revealed that among the four antibiotics tested, tobramycin had the most distinct impact on the gut microbiota composition after 24 h incubation. In the tobramycin-treated group, *Verrucomicrobiaceae* and *Erysipelotrichaceae* decreased, while *Lachnospiraceae* and *Ruminococcaceae* increased substantially. Data on the effect of the antibiotics on the fecal microbiota at phylum level are presented in Figure S1 in the supplementary materials. These data reveal that *Firmicutes*, *Verrucomicrobia*, and *Bacteroidetes* are the main contributors while also at phylum level especially tobramycin appeared to affect the bacterial composition. Tobramycin treatment resulted in an increase in the relative abundance of *Firmicutes* and an accompanying decrease in the relative abundance of *Verrucomicrobia* (Figure S1 in supplementary information).

**Fig. 3:**
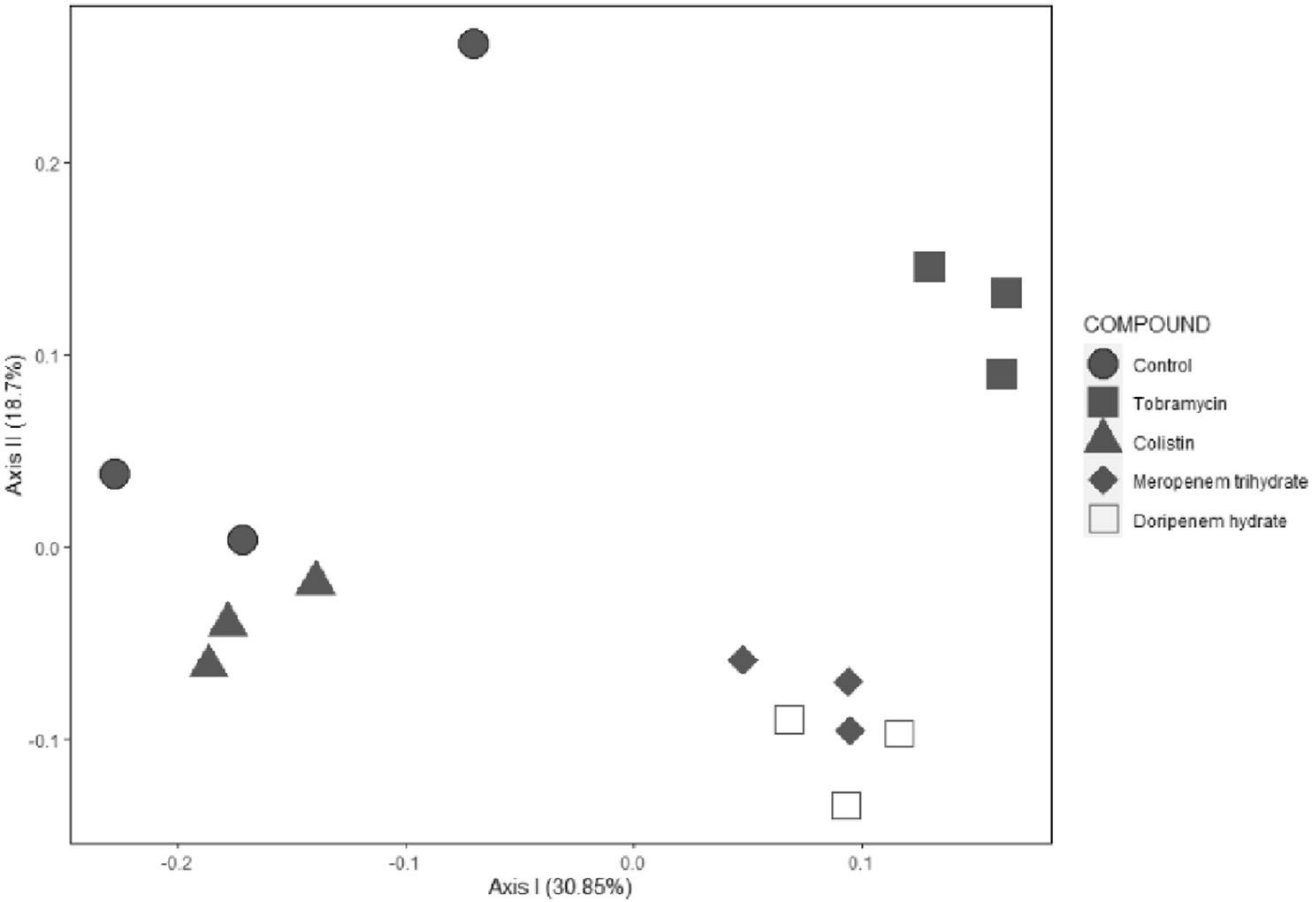
Principal coordinate analysis (PCoA) of the gut microbiota from control rat fecal samples incubated anaerobically in vitro for 24 h with or without (control) antibiotics

The effect of the antibiotics on the gut microbiome was also revealed by a principal coordinate analysis (PCoA) of Bray–Curtis distance matrix (Fig. 3). In this analysis especially the tobramycin and the carbapenems’ antibiotics (meropenem trihydrate and doripenem hydrate which clustered together), appeared different from the control group, with tobramycin also being different from the carbapenems’ antibiotics, while the colistin sulfate samples did not clearly cluster different from the control. The bacterial load of the fecal samples incubated in vitro without (control) or with in vitro antibiotic treatment, determined as gene copy numbers per gram wet weight, is presented in supplementary information Figure S2. These results show that there were no significant effects of the antibiotic treatments on the bacterial load. The alpha diversity of the samples with or without (control) in vitro antibiotic treatment (supplementary material Figure S3) showed that the tobramycin samples had a higher alpha diversity compared to the control group and the other antibiotic treatment groups (supplementary information Figure S3).

### Bile acid metabolites profiling

**Fig. 4:**
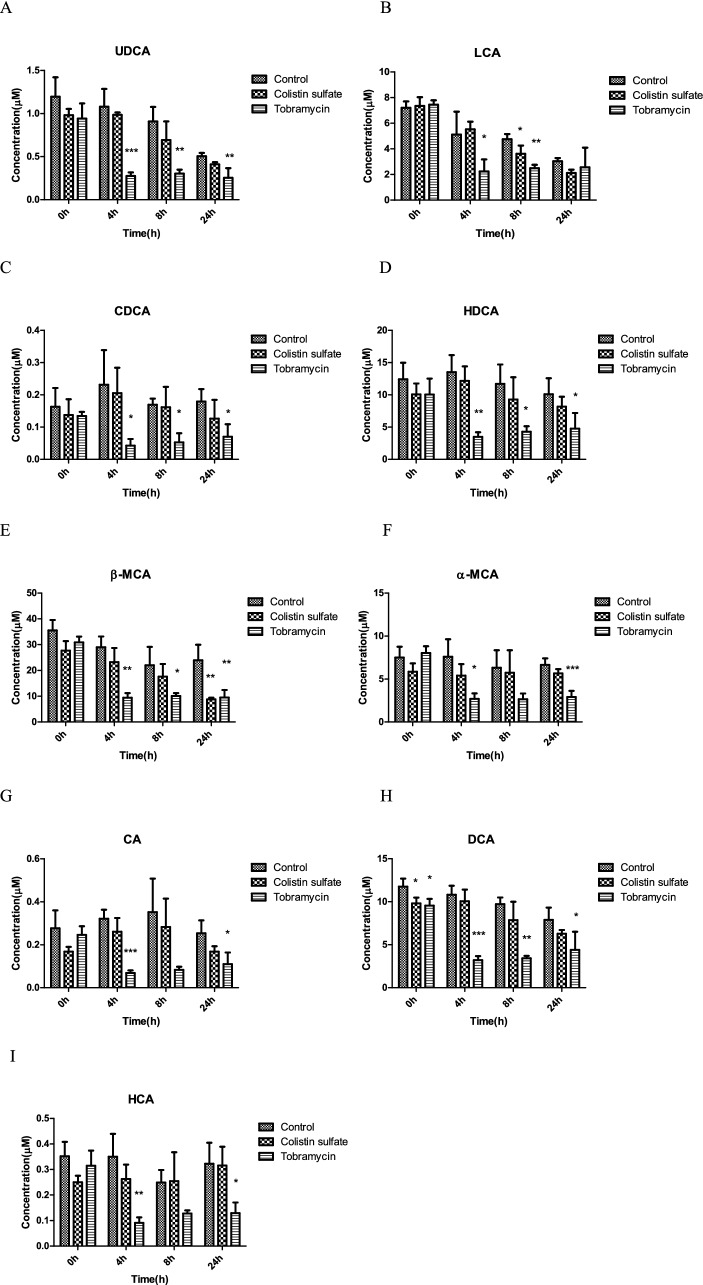
Intrinsic fecal bile acid composition and changes (A–I) over time (0 h, 4 h, 8 h, 24 h) in in vitro anaerobic rat fecal incubations with or without (control) treatment with the antibiotics colistin sulfate and tobramycin (*P < 0.05, **P < 0.01, ***P < 0.001 indicate a difference from the control without antibiotic at the corresponding time point). Results are shown as mean ± SD from three independent incubations

Based on the 16S rRNA sequencing results, tobramycin and colistin sulfate were chosen as the antibiotics to further study the effect of the antibiotics on the bile acid conversion by the gut microbiota. Colistin sulfate would represent a negative control, whereas tobramycin was expected to affect the microbiota and thus potentially also the bile acid metabolism and profile. Figure 4 shows the time-dependent bile acid profiles in supernatants collected over 24 h from anaerobic rat fecal incubations in the absence (control) or presence of the antibiotics as quantified using LC–MS/MS.

At the start of the incubations, 9 bile acids originating from untreated rat fecal samples were detected, including 6 primary bile acids (α-MCA, β-MCA, UDCA, HCA, CA, and CDCA) and 3 secondary bile acids (DCA, LCA, and HDCA). The levels of these bile acids decreased in the order: β-MCA > HDCA > DCA > LCA = α-MCA > UDCA > HCA = CA > CDCA (Fig. 4A–I). The fecal sample incubations appeared not to contain detectable levels of conjugated primary bile acids, most likely because of their efficient deconjugation (see below).

The results presented in Fig. 4A–I also reveal that upon prolonged incubation especially the incubations with tobramycin showed a significant decrease in the levels of all bile acids, while the changes in the colistin sulfate group were less pronounced and matched those observed for the control. Some bile acids, including especially UDCA, LCA, and DCA, showed a significant time-dependent decrease also in the control and colistin sulfate incubations albeit to a significantly lower extent than the decrease observed for the tobramycin incubations.

**Fig. 5:**
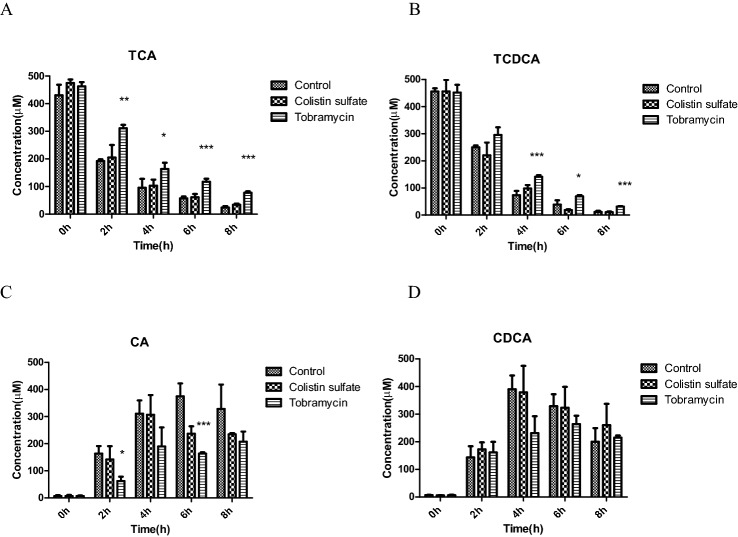
Time-dependent deconjugation of taurine conjugated bile acids to their deconjugated metabolites in in vitro anaerobic rat fecal incubations (A–D) in the absence (control) and presence of the antibiotics colistin sulfate and tobramycin (*P < 0.05, **P < 0.01, ***P < 0.001 indicate a difference from the control without antibiotic at the corresponding time point). Results are shown as mean ± SD from three independent incubations

**Fig. 6:**
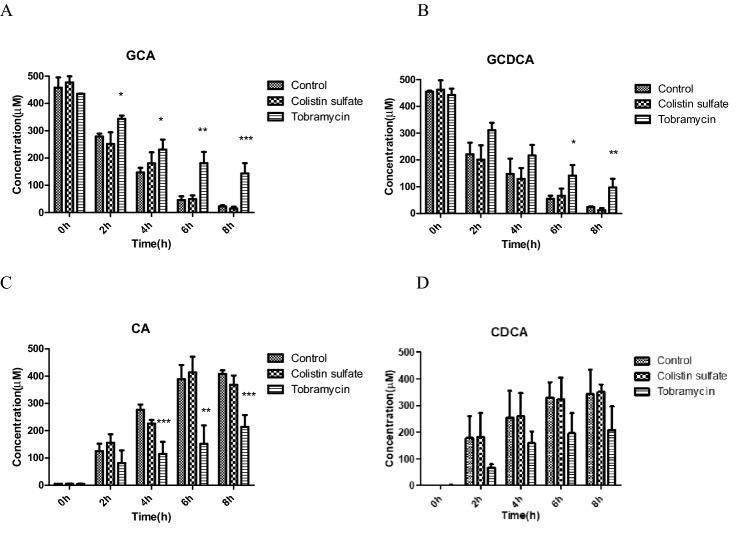
Time-dependent deconjugation of glycine conjugated bile acids to their deconjugated metabolites in in vitro anaerobic rat fecal incubations (A–D) in the absence (control) and presence of the antibiotics colistin sulfate and tobramycin (*P < 0.05, **P < 0.01, ***P < 0.001 indicate a difference from the control without antibiotic at the corresponding time point). Results are shown as mean ± SD from three independent incubations

Given that the fecal incubation did not contain detectable levels of conjugated primary bile acids, in a next series of experiments, the fecal incubations were repeated with externally added TCA, TCDCA, GCA, or GCDCA to also study the effects of the antibiotics on the potential of the fecal microbiota for deconjugation of these conjugated primary bile acids. The results thus obtained revealed deconjugation to be extremely fast, so that in these incubations, 80-fold lower concentrations of fecal slurry had to be added to allow time-dependent detection of the deconjugation. Figure 5 and 6 present the results obtained.

Deconjugation of TCA and TCDCA (Fig. 5A, B) was readily detected and accompanied by formation of their corresponding unconjugated primary bile acids CA and CDCA (Fig. 5C, D). Tobramycin significantly delayed the deconjugation of TCA and TCDCA and accompanying formation of CA and CDCA compared with the control group and the colistin sulfate group, the latter two again showing similar results.

The deconjugation of GCA and GCDCA (Fig. 6A, B) was also significantly reduced upon tobramycin treatment compared to the control and colistin sulfate groups, also reflected by relatively slower appearance of the corresponding deconjugated primary bile acids CA and CDCA (Fig. 6C, D).

To enable comparison of the in vitro effects of tobramycin on the deconjugation of conjugated primary bile acids by fecal intestinal microbiota to effects induced by tobramycin in vivo, TCA as the model compound was incubated with fecal slurry from rats that were either untreated (control) or treated for 23 days with 1000 mg/kg bw/day tobramycin in vivo.

**Fig. 7:**
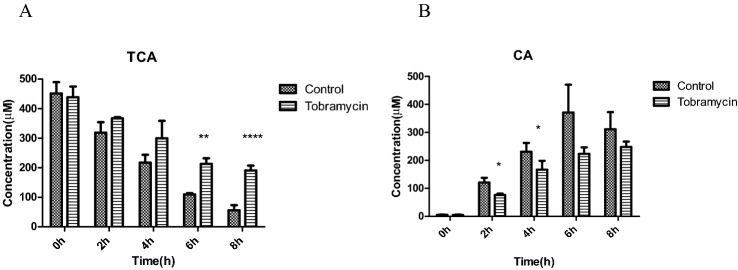
Time-dependent deconjugation of TCA (**A**) and formation of its metabolite CA (**B**) in in vitro anaerobic incubations with fecal samples from rats exposed for 23 days to 0 (control) or 1000 mg/kg bw/day tobramycin in vivo (*P < 0.05, **P < 0.01, ****P < 0.0001 indicate a difference from the control without antibiotic at the corresponding time point). Results are shown as mean ± SD from three independent incubations

Figure 7A, B presents the results of these incubations and reveals that fecal samples from rats treated for 23 days with tobramycin also show a significantly reduced activity for deconjugation of TCA to CA, as compared to fecal samples from control untreated rats.

### Effects of tobramycin on bile acid transport across a Caco-2 cell layer

In addition to an effect on TCA deconjugation, the increased fecal TCA levels observed upon in vivo treatment of rats with tobramycin (Murali et al. in preparation) may also be caused by and effect of tobramycin on bile acid reabsorption, Therefore, in addition to the in vitro studies on the effect of the selected antibiotics on microbiota composition and on bile acid metabolism by the gut intestinal fecal microbiota, additional studies aimed to characterize the effects of tobramycin on intestinal bile acid reuptake also using an in vitro model.

### WST-1 assay to determine cell viability

A WST-1 assay was performed to identify non-cytotoxic concentrations of tobramycin. The results obtained (Figure S4 in supplementary information) revealed that the Caco-2 cells were not affected by tobramycin up to concentrations of 45 mM tobramycin, the concentration selected for the transport experiments.

### Monolayer integrity

TEER values were measured before, during, and after the TCA transport experiment to check whether the TEER value of the control group and the tobramycin-treated cell layers remained unaffected during the whole experiment. The results confirmed that TEER values were consistent between the control group and tobramycin-treated Caco-2 cell layers (Figure S5 in supplementary information).

### Tobramycin inhibited TCA transport across a Caco-2 cell layer

**Fig. 8:**
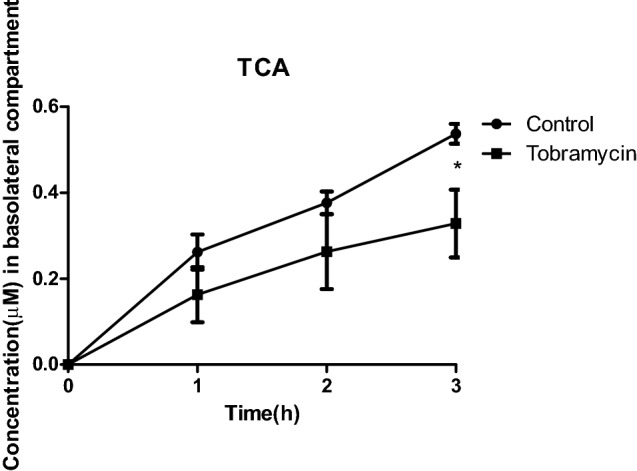
Time-dependent translocation of TCA across a Caco-2 cell layer upon pre-exposure with 45 mM tobramycin or without tobramycin (control) (*P < 0.05 indicates a difference from the control without antibiotic at the corresponding time point). Results are shown as mean ± SD from four independent experiments

Figure 8 shows the time-dependent transport of TCA across the Caco-2 cell layer upon pre-treatment of the Caco-2 cells with tobramycin as compared to the control. The results reveal that pre-treatment of the Caco-2 cells with 45 mM tobramycin significantly inhibited the translocation of TCA across the Caco-2 cell layer.

## Discussion

In vivo studies in which experimental animals are exposed to antibiotics have revealed potentially substantial effects of antibiotics on the host fecal and blood serum metabolome especially on bile acid homeostasis (Behr et al. 2018a). In these in vivo studies increased fecal levels of conjugated bile acids like, for example, TCA were frequently observed (Behr et al. 2019). In theory, such increased fecal levels of conjugated bile acids upon exposure to antibiotics may be due to different underlying modes of action including especially an effect on bile acid metabolism by the intestinal microbiota and/or an effect on the process of bile acid reabsorption. Given that 95% of the bile acids are reabsorbed into the systemic circulation, it can be expected that an inhibition of this process will readily result in increased fecal bile acid levels, reflecting a potential disturbance of bile acid homeostasis.

Thus, in the framework of developing new approach methodologies (NAMs) replacing animal testing, the aim of the present study was to evaluate two selected in vitro models for studying effects of antibiotics on intestinal bile acid homeostasis and obtain insight in the mode(s) of action underlying the in vivo effects of the antibiotics on bile acid homeostasis. To this end, anaerobic fecal incubations as an in vitro model for studying effects of the antibiotics on gut microbiota and their bile acid metabolism as well as Caco-2 cell layers in a Transwell model as an in vitro approach to study intestinal bile acid reabsorption were evaluated.

16S rRNA sequencing analysis was used to profile the composition of the intestinal microbiome after exposure with antibiotics (tobramycin, colistin sulfate, meropenem trihydrate, and doripenem hydrate). Among the antibiotics tested tobramycin appeared to have the most obvious influence on microbial composition at family level, with reduced contributions of *Verrucomicrobiaceae* and *Erysipelotrichaceae* and increased amounts of *Lachnospiraceae* and *Ruminococcaceae*. *Verrucomicrobiaceae* belong to the *Verrucomicrobia* which is a phylum of Gram-negative bacteria (Devos 2014; Szuróczki et al. 2020). *Erysipelotrichaceae* are part of the *Firmicutes* a phylum of Gram-positive bacteria (Kaakoush 2015). *Lachnospiraceae* and *Ruminococcaceae* also belong to the *Firmicutes* (Gu et al. 2013). The overall increase of *Firmicutes* at the expense of *Verrucomicrobia* upon tobramycin treatment is in accordance with its activities as an antibiotic affecting mainly Gram-negative bacteria (Neu 1976). In an accompanying in vivo study (Murali et al. in preparation), tobramycin and colistin sulfate were also tested in 28 day oral toxicity studies at BASF. This enables comparison of the effects now detected in the in vitro anaerobic fecal incubations to those obtained in vivo. Comparison of the in vitro and in vivo 16S rRNA analysis results, revealed that both in vitro and in vivo tobramycin has the most substantial effects, while the effects of colistin sulfate appeared limited. Similar to the PCoA of the present in vitro studies, an in vivo PCoA analysis of the 16S RNA data of fecal samples obtained upon 28-day treatment of rats with these antibiotics demonstrated that the tobramycin group separated from the control group, while the colistin sulfate samples did not clearly cluster different from the control. Furthermore, in control fecal samples in both the in vivo and in vitro study, the dominant families were *Verrucomicrobiaceae*, *Porphyromonadaceae* followed by *Lachnospiraceae* and *Ruminococcaceae*, while also the effect of tobramycin appeared to be similar resulting in a reduction of *Verrucomicrobiaceae* and an increase in *Lachnospiraceae* (Murali et al. in preparation)*,* again corroborating the consistency between the in vivo and in vitro study. In both the in vitro and in vivo study, colistin sulfate treatment showed limited effects. The in vitro data revealed that meropenem trihydrate and doripenem hydrate were different from controls as illustrated by similar effects on *Erysipelotrichaceae, Lactobacillaceae*, and *Ruminococcaceae* as observed for tobramycin but less distinct.

The in vivo studies also reported effects on the fecal and plasma metabolome with substantial effects of tobramycin treatment on especially fecal levels of the bile acid TCA. Therefore, further experiments of the present paper focused on tobramycin and the potential modes(s) of action underlying this substantial increase in fecal TCA levels upon tobramycin treatment. In in vitro anaerobic incubations with and without extra added, external bile acids tobramycin treatment significantly reduced degradation of conjugated bile acids including TCA, while in incubations with colistin sulfate, the time-dependent bile acid degradation matched that observed in the control without added antibiotics. These results revealed that tobramycin inhibits TCA deconjugation, an observation that was corroborated by incubating TCA with fecal samples obtained from control rats and with fecal samples from the rats treated for 23 days with tobramycin, which revealed significantly reduced rates of TCA deconjugation by fecal samples from tobramycin-treated rats. This in vitro observation provides insight in a potential mode of action underlying the in vivo observed increases in fecal TCA levels, and indicates that upon treatment with tobramycin, the intestinal microbiota become less efficient in deconjugation of this primary bile acid.

It is of interest to note that in the in vitro incubations without extra added external bile acids, no conjugated bile acids were detected, while the concentrations of all non-conjugated bile acids appeared to decrease in time. This first observations pointed at the highly efficient deconjugation by the fecal microbiota, while the latter observation may best be explained by further conversion of these non-conjugated bile acid by the intestinal microbiota to products not included in the LC–MS/MS-based analysis. For example, the primary bile acids α-MCA and β-MCA known to be especially present in rodents can be converted to secondary bile acids like ꞷ-MCA (Chiang 2017), a bile acid not included in the LC–MS/MS method applied. The time-dependent reduction in bile acids may also be ascribed to metabolism of bile acids regarding pathways enabling their use as energy source (Ma and Patti 2014). This latter explanation would be consistent with the upregulation of *Firmicutes* upon tobramycin treatment, because some literature studies report on increased energy harvest induced by increased levels of *Firmicutes* (Figure S1 in supplementary information) (Turnbaugh et al. 2006).

In addition to inhibition of TCA deconjugation upon treatment of the intestinal microbiota by tobramycin, it was also investigated whether the increased fecal levels of TCA upon tobramycin treatment of rats in vivo may be due to an effect on intestinal reuptake. Given that 95% of bile acids are actively reabsorbed, a limited effect on this reabsorption involved can be expected to substantially increase fecal levels of bile acids.

Upon pre-exposure of the Caco-2 cell monolayers with tobramycin, the transport of TCA across the intestinal monolayer was significantly reduced. The reduction transport activity upon pre-exposure may be related to the fact that tobramycin, being an aminoglycoside antibiotic, is able to facilitate read-through at stop codons at the ribosomes, thus reducing the synthesis of normal function proteins (Prokhorova et al. 2017; Wangen and Green 2020). Hence, it is possible to hypothesize that the expression and thus activity of the apical sodium-dependent bile acid transporter (ASBT) was inhibited by tobramycin at the level of protein synthesis at the ribosomes. In addition, increased bile acid efflux from the liver and/or enhanced bile acid synthesis—the latter resulting from the induction of Cyp7a1 expression in the liver upon depletion of gut microbiota by antibiotics (Schneider et al. 2021)—may contribute to the increased intestinal and subsequent fecal TCA levels. Thus, the increase in fecal TCA levels can be ascribed to a reduction in deconjugation capacity resulting from changes in the intestinal microbiome, an effect on the reuptake via effects on synthesis and activity of the transport proteins involved, and/or an effect on de novo bile acid synthesis and efflux from the liver.

In addition to the effects of antibiotics on bile acid homeostasis at the levels investigated in the present study, it was reported that depletion of gut microbiota by antibiotics may result in induction of Cyp7a1 expression in the liver, thereby enhancing bile acid synthesis (Awoniyi et al. 2022; Schneider et al. 2021). Development of a new approach methodology (NAM) to capture these effects is an interesting topic for future studies. Such studies may include identification of the antibiotic-related changes in host blood or plasma bile acid metabolome profiles. Earlier in vivo studies already reported that antibiotics, including lincomycin, clindamycin, roxithromycin, vancomycin (Behr et al. 2019), and also tobramycin (Murali et al. in preparation), affected host blood or plasma metabolome patterns including bile acid concentrations resulting in an increase in the ratio of conjugated versus unconjugated bile acids. This finding is in line with results reported by Schneider et al. (Schneider et al. 2021). This relative increase in the ratio of conjugated versus deconjugated bile acids may provide a rationale for a potential effect on the expression of liver enzymes involved in bile acid synthesis. Including the effects observed in the present study in a physiologically based kinetic (PBK) model for bile acid homeostasis, and use of the in vitro systems to define the relevant PBK model parameters for uptake and metabolism, may provide an NAM to elucidate to what extent the inhibition of bile acid deconjugation and reuptake by tobramycin in the intestine may affect systemic bile acid levels and provide a rationale for the effect on bile acid synthesis in the liver.

The in vitro studies of the present study also provide a basis to use the respective models to study the effect of combined antibiotic exposure (Worthington and Melander 2013). This is of relevance given that in daily practice, often cocktails of broad spectrum antibiotics are applied. Use of in vitro models facilitates testing of a wider range of combinations providing insight in potential combination effects and/or enabling the definition of priorities for potential in vivo studies.

Altogether, the results of the present paper provide a first proof-of-principle that in vitro studies can be of use to elucidate the effect of in vivo antibiotics, and thus also of other xenobiotics, on the intestinal microbiota and their bile acid metabolism as well as on reuptake of bile acids, all resulting in effects on bile acid homeostasis.

## Supplementary Information

Below is the link to the electronic supplementary material.

Supplementary file1 (DOCX 269 KB)
