# Supplementary material for: In vitro models to detect in vivo bile acid changes induced by antibiotics
Source: Arch Toxicol. 2022 Sep 8;96(12):3291–303. doi: 10.1007/s00204-022-03373-4 (PMC9584874; doi:10.1007/s00204-022-03373-4)
Supplement: Supplementary file 1 — Supplementary file1 (DOCX 269 KB) [file 204_2022_3373_MOESM1_ESM.docx]

***In vitro* models to detect *in vivo* bile acid changes induced by antibiotics**

Nina Zhang^1^, Jingxuan Wang^1^, Wouter Bakker^1^, Weijia Zheng^1^, Marta Baccaro^1^, Aishwarya Murali^2^, Bennard van Ravenzwaay^2^, Ivonne M. C. M. Rietjens^1^

^1^Division of Toxicology, Wageningen University and Research, Stippeneng 4, 6708 WE

Wageningen, The Netherlands

^2^BASF SE, 67056 Ludwigshafen, Germany

*Corresponding author: Nina Zhang: nina1.zhang@wur.nl

**Supplementary information**

**Table S1.** conversion *in vivo* antibiotics does level to *in vitro* antibiotics concentration level

| Name | *In vivo* high dose level | *In vitro* high concentration |
| --- | --- | --- |
| Colistin sulfate | 100 mg/kg bw/d | 2 mM |
| Tobramycin | 1000 mg/kg bw/d | 45 mM |
| Meropenem trihydrate | 300 mg/kg bw/d | 15 mM |
| Doripenem hydrate | 1000 mg/ kg bw/d | 50 mM |


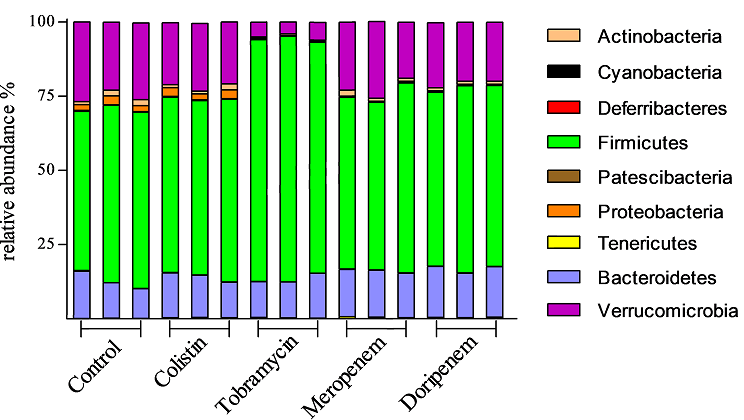


**Figure S1.** Phylum level of the 24h rat fecal samples with or without (control) *in vitro* antibiotic treatment, it showed as (mean± SD, n=3)

**
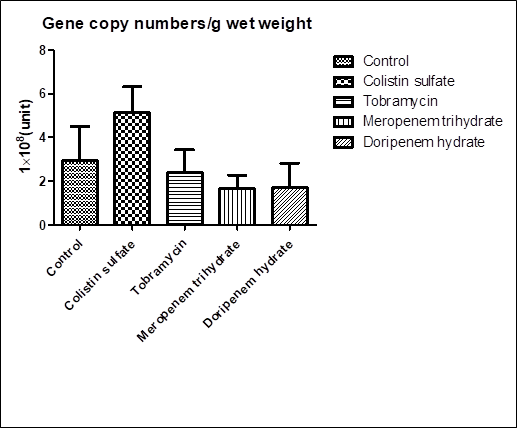
**

**Figure S2.** Bacterial load of the 24h rat fecal samples with or without (control) *in vitro* antibiotic treatment, performed as gene copy numbers per gram of wet weight of fecal slurry(mean ± SD, n=3)


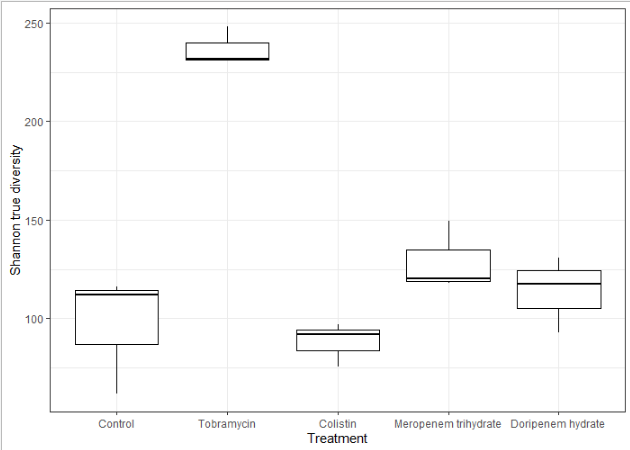


**Figure S3.** Alpha diversity of the 24h rat fecal samples with or without (control) *in vitro* antibiotic treatment, it showed as (mean ± SD, n=3)

**Figure S4.** WST-1 assay defined the non-cytotoxic concentration of tobramycin, y axis represented the percentage of cell viability, results are shown as (mean ± SD, n=3)

**Figure S5.** TEER values before (MEM medium), during (transport medium) and after the TCA transport experiment for the control and tobramycin treated Caco-2 cell layers during pre-exposure study. Results are shown as (mean ± SD, n=4).
